# Nanoscale Polishing Technique of Biomedical Grade NiTi Wire by Advanced MAF Process: Relationship between Surface Roughness and Bacterial Adhesion

**DOI:** 10.3390/jfb14040177

**Published:** 2023-03-23

**Authors:** Se Rim Jang, Il Won Suh, Lida Heng

**Affiliations:** 1 Division of Mechanical Design Engineering, College of Engineering, Jeonbuk National University, 567, Baekje-daero, Jeonju-si 54896, Republic of Korea; tpfla6020@gmail.com; 2 Department of Bionanotechnology and Bioconvergence Engineering, Graduate School, Jeonbuk National University, Jeonju 561-656, Republic of Korea; ilwon1562@gmail.com

**Keywords:** bacterial adhesion, biomedical grade NiTi wire, nanoscale polishing technique, MAF process, smooth surface polish, balloon angiography

## Abstract

Nitinol (NiTi), an alloy of nickel and titanium, wires are an important biomedical material that has been used in catheter tubes, guidewires, stents, and other surgical instruments. As such wires are temporarily or permanently inserted inside the human body, their surfaces need to be smoothed and cleaned in order to prevent wear, friction, and adhesion of bacteria. In this study, NiTi wire samples of micro-scale diameters (i.e., Ø 200 μm and Ø 400 μm) were polished by an advanced magnetic abrasive finishing (MAF) process using a nanoscale polishing method. Furthermore, bacterial adhesion (i.e., *Escherichia coli* (*E. coli*), and *Staphylococcus aureus* (*S. aureus*)) to the initial and final surfaces of NiTi wires were investigated and compared in order to assess the impact of surface roughness on bacterial adhesion to the surfaces of NiTi wires. The finding revealed that the surfaces of NiTi wires were clean and smooth with a lack of particle impurities and toxic components on the final surface polished using the advanced MAF process. The surface roughness *Ra* values of the Ø 200 μm and Ø 400 μm NiTi wires were smoothly enhanced to 20 nm and 30 nm from the 140 nm and 280 nm initial surface roughness values. Importantly, polishing the surfaces of a biomedical material such as NiTi wire to nano-level roughness can significantly reduce bacterial adhesion on the surface by more than 83.48% in the case of *S. aureus*, while in the case of *E. coli* was more than 70.67%.

## 1. Introduction

Temporary and permanent orthopedic implants (e.g., stent wires, artificial hips, knees, and shoulder joints) have been used in the orthopedic industry to serve functions in the physical body throughout the lifespans of patients [1,2,3,4]. These orthopedic implants are generally made of nitinol (NiTi) [5], titanium [6], stainless steel 316L [7], or ceramic [8]. Among them, a stent wire is one of the most widely used components in medical applications. A stent is a cylindrical precision medical device that is typically made of nitinol (NiTi), an alloy with a near-equiatomic composition of nickel (Ni) and titanium (Ti). NiTi wires are one of the most suitable biomedical materials, which have been widely used in the medical industry due to their superior mechanical characteristics such as shape recoverability, biocompatibility, low density, high elastic modulus, high strength, durability, and corrosion resistance [5,9,10]. They can be implanted into a lumen (e.g., blood vessels, colon, biliary tract, heart, and brain) for treatment when a thrombus or tumor is present [11,12,13]. Since they are implanted further inside the patient’s body, their surfaces need to be cleaned and smoothed [14]. In addition, there is a high demand to decrease impurities and material toxicology on their surfaces in order to avoid health risks [15]. When implanting a stent with high surface roughness, damage can occur to the lumen and the inner walls of blood vessels to form a thrombus, which causes restenosis [16]. In addition to the problems with the surface roughness of stent wires, there is a major problem that occurs on the implant surface of NiTi stent wires, known as an infection-related implant (IRI). IRIs cause the evolution of bacteria at the implantation site as a result of bacterial adhesion to an implant surface [17,18]. This problem is caused by gram-positive bacteria (GPB) that have a significant impact on patient health, such as inflammatory reactions, lethal complications, and even death [19,20]. Especially for nonvascular and coronary stents, fluids that remain between stents and organs can cause the formation of a biofilm [21]. The growth of a biofilm can cause infections, leading to strong adverse reactions between the stent and tissue (e.g., chronic pulmonary infection, cystic fibrosis, and chronic wounds) [22,23]. Therefore, reducing bacterial adhesion to biomedical material surfaces such as stent wires, is crucial for enhancing the success rate of stent insertion surgery, potentially resulting in a dramatic reduction in implant-related infections.

Generally, bacterial adhesion to the surface of biomedical materials (BMMs) can be directly affected by material surface features such as topography, roughness, and surface charge [22,24]. Among these features, the physical and chemical characteristics of the material directly affect bacterial adhesion. Micro/nano-scale topographical features are an important parameter for bacterial adhesion [25,26]. Many studies have demonstrated that different kinds of bacteria (i.e., *Escherichia coli, Staphylococcus aureus*, *Staphylococcus epidermidis*, *Propionibacterium acnes*, and so on) favor adhesion on a micropatterned surface of the biomedical materials [27,28,29,30]. Lu et al. [29] have studied the impacts of different surface roughness values on the bacterial adhesion (type: *Staphylococcus aureus*) on the surface of bio-ceramic joint implants. In his study, the bio-ceramic joint implants were used as the samples (size: 30 × 30 × 5 mm^3^). The smooth surface of the sample was achieved from 205 nm to a nano-scale surface roughness of 1.1 nm *Ra* via the precision grinding and wheel polishing process. He found that achieving a nano-scale surface roughness on a bio-ceramic surface is an efficient way to reduce bacterial adhesion. Zhao et al. [31] have applied the ion implantation technique using (N^+^, O^+,^ and SiF_3_^+^ ions) to achieve a smooth surface of stainless steel disk samples and also to reduce the bacterial adhesion (type: *Staphylococcus epidermidis* and *Staphylococcus aureus*) on the surface of these samples. He concluded that the ion implantation technique was feasible for reducing bacterial adhesion on the surface of samples. Wu et al. [32] applied the anti-bacterial Ti–Cu–N coating process on the surface of titanium implant samples. He concluded that the anti-bacterial Ti–Cu–N coatings were successfully prepared on titanium via sputtering-deposited Ti–Cu films at a temperature of 900 °C within 3 h. As a result, this method can improve the anti-bacterial properties on the surface of titanium implant samples. The recent advances in surface modification techniques (i.e., ultrasonic nanocrystal surface modification, electrophoretic deposition, ion-implantation, glow discharge plasma, thermal spraying, and so on) for enhancing the biocompatibility of biomedical alloys (i.e., stainless steel, Co-Cr, and Ti alloy) have been reviewed by Thakur et al. [33]. These methods can prevent corrosion and improve the anti-bacterial activity of biomedical implants of various materials such as Ti, stainless steel, and Co-Cr alloy.

However, these techniques are mainly applied to the surface processing of millimeter-scale biomedical components. However, they cannot be effectively applied for processing the surface of micro-scale components, such as NiTi stent wires, due to their processing limitations with a micro-scale component. Generally, there are many effective techniques that can be used to achieve ultra-smooth surfaces of biomedical materials that also can prevent implant-associated infections. These techniques include magnetic abrasive finishing (MAF) and abrasive flow machining (AFM). Hitomi et al. [34] established an effective surface finishing process for reducing the surface roughness of Co-Cr alloy femoral knee components by the MAF process. Yahya et al. [35] used the abrasive flow finishing process for reducing the surface roughness of hip joint implants, such as the SS 316L femoral head product, using the antithesis replica fixture procedure. Satish et al. [36] developed the nano-finishing of a freeform surface component by a rotational-magnetorheological abrasive flow finishing (R-MRAFF) process to polish a hip joint implant. Their results revealed successful improvement of the surface quality of these products by virtue of a smooth surface. However, they could only achieve smooth surfaces for large and millimeter-scale components. These important techniques cannot be effectively used to fabricate high surface accuracy with a low surface roughness of micro-scale features or components, such as NiTi stent wires. This is because the stent wires themselves are very easily weakened, broken, or damaged by these processes due to their micro-scale diameters. The structural integrity of microscale wire components can be compromised by smoothing methods that remove significant quantities of material in comparison to their diameters. Moreover, the installation of stent wires into the processing equipment of these processes is very difficult due to their micro-scale size, making it difficult for them to be fixed or moved during their polishing processes. In addition, these processing methods create problems when a stent is inserted because the structural and tensile strengths of the nonuniform wires can complicate the insertion of the stent and subsequent removal of components following balloon angiography stent placement. In other words, the uniformity of the wires in a stent directly impacts the surgical procedure, the long-term function and geometry of the stent, and bacterial infection-related consequences of stent placement. Currently, the surfaces of stent wires are processed by surface treatment techniques (e.g., plasma source ion implantation, laser polishing, and coating) [37,38,39]. Manu et al. [39] evaluated the effects of surface ion implantation characteristics of stent archwires (i.e., NiTi and TiMo). They found that the surface quality values of NiTi and TiMo were significantly reduced to respective values of 330.87 and 236.35 nm from the initial *Ra* values of 795.95 and 463.28 nm by this process. Park et al. [40] used a laser polishing process to polish the surface of implant materials, particularly a biliary NiTi stent. They revealed that the surface abrasion Ra of the NiTi stent was reduced by 34–64% after the laser polishing technique. A coating process is another technique that can be used to enhance corrosion resistance and reduce the surface texture of NiTi wires, contributing to increased corrosion avoidance of NiTi wires [41]. Natalia et al. [42] studied the characterization of ZrO_2_ ceramic nano-coatings on NiTi wires. In their study, a ZrO_2_ coating was accumulated on the surface of NiTi wire by cathodic electrodeposition with pulses and two different solutions (ZrO(NO_3_)_2_ and ZrOCl_2_). The surface roughness *Ra* value of NiTi wires was reduced to 2.1 ± 0.3 nm by ZrO_2_ coating within a 1200 sec processing time. According to the results of these researchers, these processes are feasible for successfully achieving stent wires with a smooth surface. However, each of these processes has limitations that can cause serious side effects on human health, reduced biocompatibility, processing time, and energy consumption. As reported by Solheid et al. [43], during the laser polishing process, high temperatures occur on the polishing zone at the surface of the workpiece and can create extended areas of heat-affected tissue with resulting microstructural changes that significantly influence the biocompatibility of a thin stent wire. Lopes et al. [44] have found problems with ZrO_2_ coatings, particularly that the production of ZrO_2_ nano-coatings is difficult due to their reduced dimensions. Moreover, this process requires long processing for up to 1200 s to achieve NiTi stent wires with smooth surfaces, greatly increasing the costs to produce these products. The ion implantation process has major limitations of very expensive equipment [45,46], use of toxic materials (e.g., phosphine and arsine), high voltage accelerators, and high currents that can affect human health and safety [47].

To overcome the limitations of these processes, an innovative method is required to create high-precision microscale NiTi stent wires with ultra-smooth surfaces that do not result in adverse side effects. In this study, an advanced magnetic abrasive finishing process using a nanoscale polishing technique is used for polishing the surfaces of NiTi wires. It is a method that applies eco-friendly abrasive polishing, called magnetic micro-abrasive polishing. A mixture of grape seed oil, micron-sized pure iron (Fe) powder, and polycrystalline diamond (PCD) micro-tools was used to fill the space between the north and south magnetic poles to remove the unevenness and particle impurities from the cylindrical surfaces of NiTi wires. By controlling the abrasive polishing process’s cutting action on the surfaces of NiTi stent wires, ultra-smooth and clean surfaces of the NiTi wires were achieved.

In this study, a novel advanced magnetic abrasive finishing (MAF) process using the nanoscale polishing technique has been used for achieving an ultra-smooth surface of biomedical grade NiTi wires that have micro-scale diameters (i.e., Ø 200 μm and Ø 400 μm). The effects of critical input parameters (i.e., rotational speeds of the magnetic pole, grain sizes of PCD micro-tools, and polishing times) on achieving an ultra-smooth surface with a nano-level surface roughness *Ra* of NiTi wires were evaluated. In addition, two kinds of bacteria (i.e., *Escherichia coli* and *Staphylococcus aureus*), which are the most commonly adhere on the surface of biomedical materials, were chosen for the bacterial adhesion tests. The relationship between the nanopatterned surfaces of NiTi wires and bacterial adhesion (i.e., *Escherichia coli* and *Staphylococcus aureus*) were studied, and the adhesion mechanism of the bacteria on such surfaces was analyzed.

## 2. Materials and Methods

### 2.1. Materials Selection

**Figure 1:**
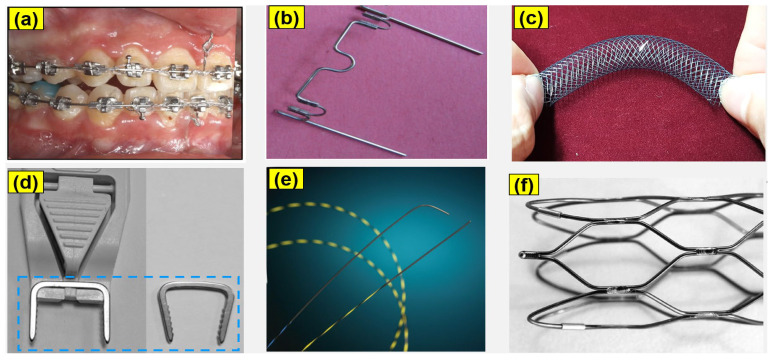
Actual implementation of biomedical grade NiTi wires used in orthopedic implants: (**a**) archwire orthodontics [48], (**b**) palatal arch (Data obtained from Petrini et al. [49], CC-BY 4.0), (**c**) biliary stent, (**d**) compression staple [50], (**e**) tip guidewire [51], (**f**) coronary stent [52]. Reused with permission from Elsevier.

Biomedical grade NiTi wires in micro-scale diameter (i.e., Ø 200 and Ø 400 μm) are widely used in orthopedic applications. They were used as the samples in this study. These wires were produced by Kellogg’s Research Labs, New Boston, NH, USA. They had original surface roughness *Ra* values of 280 nm for the Ø 400 μm wires and 140 nm for the Ø 200 μm NiTi wires. The chemical compositions of the NiTi wires used in the study were obtained using FE-SEM (Model: JSM-7100F) EDS spectrum analysis. Table 1 shows the chemical compositions of the Ø 200 and Ø 400 μm NiTi wires. The actual implementation of medical grade NiTi wires used in orthopedic implants is shown in Figure 1: (a) archwire orthodontics, (b) palatal arch, (c) biliary stent, (d) compression staple, (e) tip guidewire, and (f) a coronary stent.

### 2.2. Bacterial Adhesion Tests

**Figure 2:**
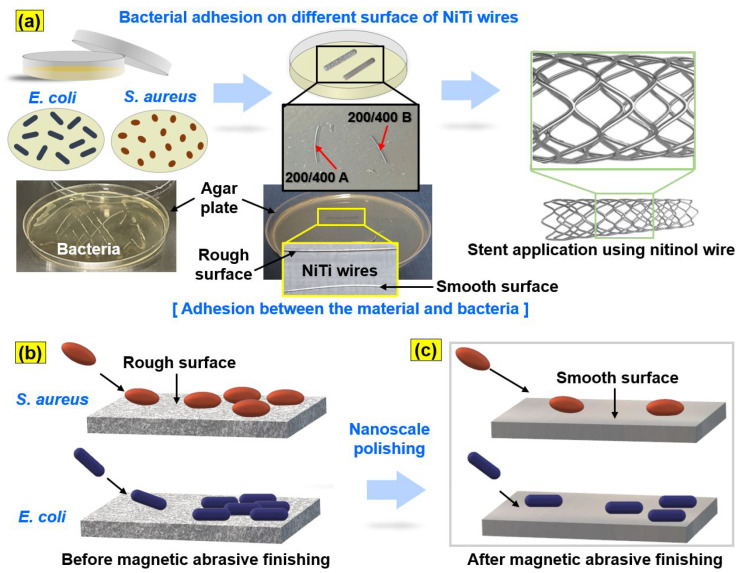
Schematic illustration of the experiment preparation and mechanism of surface bacterial adhesion of NiTi wire materials: (**a**) bacterial adhesion experiment preparation, (**b**) bacterial adhesion on the rough surface of NiTi wire, and (**c**) on the smooth surface of NiTi wire after nanoscale polishing.

Two kinds of bacteria (i.e., *Escherichia coli* and *Staphylococcus aureus*) are the most commonly adhered or attached to the surface of biomedical materials, a leading cause of mortality, especially in immunocompromised patients [53]. Therefore, they have been chosen for the bacterial adhesion tests in this study. The test preparation and mechanism of bacterial adhesion to the surface of NiTi wire are shown in Figure 2. All samples were washed using ethanol and sterilized under UV for 24 h. In this study, *Escherichia coli* (*E. coli*; gram-negative, ATCC 52922 strain, Manassas, USA) and *Staphylococcus aureus* (*S. aureus*; gram-positive, ATCC 29231 strain, Manassas, USA) were incubated in lysogeny broth (LB) medium using a shaking incubator at 37 °C for 24 h. The densities of the two bacteria were calculated from UV spectrophotometry at 680 nm (optical density ≈ 0.9). All NiTi wire samples were cut to 1.5 cm on a typical Luria Bertani (LB) agar plate, and the bacterial suspension was transferred to the samples. After incubation for 24 h, 2.5% of glutaraldehyde was applied to fix the bacteria. Then, the bacteria were evaporated using different concentrations of ethanol (i.e., 20, 30, 50, 80, and 100%) for 15 min each. Next, the samples were dried for one day at room temperature. Figure 2b shows the mechanism of large amounts of bacterial adhesion on the rough surface of a NiTi wire. Figure 2c shows the reduced amounts of bacterial adhesion on the smooth surface of the NiTi wire after nanoscale polishing with the advanced MAF process.

### 2.3. Experimental Setup of the Advanced MAF Process

**Figure 3:**
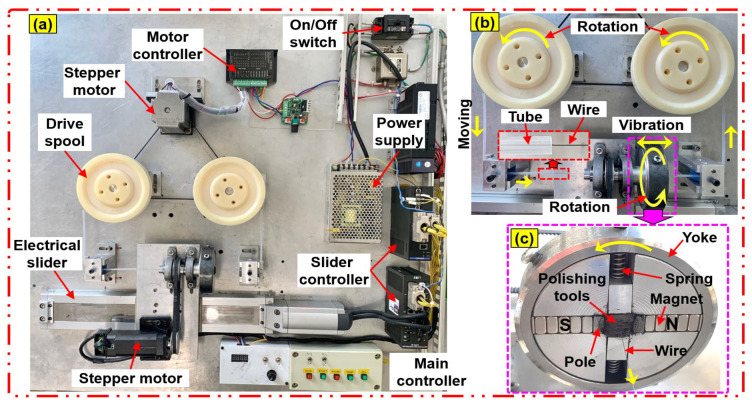
(**a**) Photograph of advanced MAF process setup, (**b**) enlarged view of vibration/rotation system of the magnetic pole, and (**c**) front view of the polishing part.

**Figure 4:**
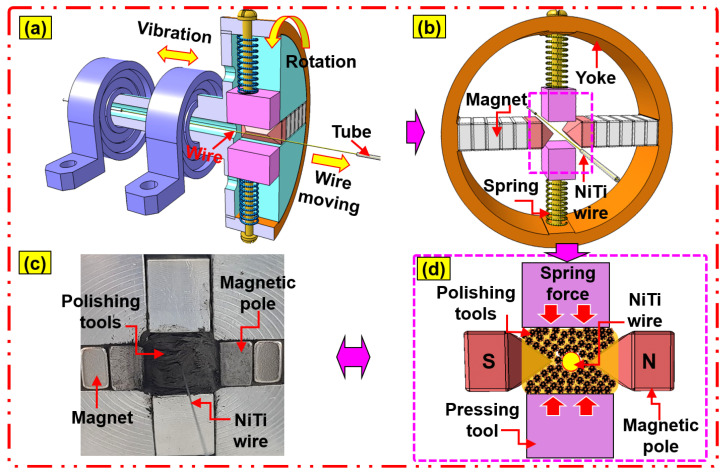
Polishing procedure of the advanced MAF process: (**a**) the NiTi wire moving inside the polishing area, (**b**) compression springs applied to the magnetic abrasive tools, (**c**,**d**) respective image and illustration views of the nano polishing principle acting on the NiTi wire.

In this study, the surfaces of NiTi wires were polished through the advanced magnetic abrasive finishing (MAF) process using a nano-scale polishing technique. The experimental setup of the magnetic abrasive finishing process for NiTi wires is illustrated in Figure 3. The advanced MAF experimental setup using the nanoscale polishing technique is composed of three main systems, (i) wire movement, (ii) magnetic pole rotation, and (iii) magnetic pole vibration. The wire movement system is composed of a stepper motor, two drive spools, a motor controller, and a silicone tube and is used to move NiTi wire inside the abrasive tools. Each end of the NiTi wire was connected to a rotating drive spool that slowly moved the wire inside the abrasive tools at a certain speed set by a motor controller. A silicone tube with an inner diameter of 2.0 mm was used to protect the cylindrical surface of the NiTi wire from wear and friction during wire movement. The magnetic pole rotating system is composed of a steel yoke, an aluminum chuck, two compression springs, two magnetic poles, two sets of permanent magnets (size: 20 × 10 × 10 mm), a mixture of polishing tools, NiTi wire, a stepper motor, and a motor controller. The motor rotates the chuck along the wire axis to machine the micro-scale diameter NiTi wire material surface with a belt; the chuck fixes a circular yoke and arranges the magnetic components in a line; the permanent magnets are attached to a flat area of the circular yoke; the pole tips are attached with magnets on each side at a pole gap of 5 mm to avoid collision with the wire, and the magnetic pole rotation system is set on the electric slider vibration system. The magnets are attached to a circular yoke to form a closed magnetic field effectively concentrated by the poles. The magnetic pole vibration system is used to generate vibration of the magnetic pole, and it includes an electric slider, slider controller, and a power supply. It can generate the vibrating action of the magnetic pole from 0 Hz to 20 Hz, which can be adjusted by a slider controller. The polishing procedure of the advanced MAF process is illustrated in Figure 4: (a) the NiTi wire moving inside the polishing area, (b) the compression springs applied to the magnetic abrasive tools, (c) and (d) the respective image and illustration views of the nano polishing principle acting on the NiTi wire. In this study, the magnetic micro-abrasive polishing (MMAP) mixture was fabricated from Fe micron powder, PCD micro-tools, and grape seed oil (GSO). The MMAP mixture filled the gap between the north and south magnetic poles attached to permanent magnets (Nd-Fe-B type) (see Figure 4c). Due to the opposite poles (N-pole and S-pole) of the two permanent magnets, the paths of the magnetic field bunch together between the two poles, resulting in the generation of a high magnetic flux density within the gap of the magnetic poles (see Figure 4d). Due to the high magnetic flux density, the abrasives inside the gap of the magnetic poles strongly attract each other, forming a polishing brush for removing the rough structure and impurity particles from the surfaces of the NiTi wire sample. During the nanoscale polishing process, the NiTi wire sample moves slowly at a feed rate of 10 mm/s inside a rotating/vibrating MMAP (1200 rpm/10-Hz). This generated relative friction between the PCD micro-tools and the surface of the NiTi wire. The relative friction causes the polishing/cutting action of the PCD micro-tools on the surface of NiTi wire, reducing rough structures (e.g., unevenness, high peaks) from the surface of the NiTi wire in the form of nano-chips. Using this procedure, an ultra-smooth and clean surface of NiTi wire was achieved by the advanced MAF process using nanoscale polishing.

The detailed experiment conditions of an advanced MAF process for biomedical grade NiTi wire samples are listed in Table 2. The samples have lengths of 250 mm with different diameters of Ø 200 μm and Ø 400 μm. The MMAP solution was produced by mixing Fe micron powder (Fe#200): 0.8 g, PCD micro-tools: 1.0 g, and grape seed oil (GSO): 400 µL. The important parameters that have an influence on the experiment are (1) rotational speeds of the magnetic pole (600, 1200, 1800 rpm), (2) grain sizes of PCD micro-tools (1, 3, 6 μm), and (3) polishing times (0, 30, 60, 90, 120, 150 s) were analyzed to compare and evaluate the NiTi wire polishing characteristics. At every 30 s of polishing time, the surface roughness *Ra* values of the NiTi wire sample were measured using a contact-type surface roughness measuring instrument (Mitutoyo SJ-400). The FE-SEM and EDS mapping methods were used to directly characterize the surface nano-structure, bacterial adhesion, and chemical compositions of the NiTi wires.

### 2.4. FE-SEM Observation and EDS Analysis

In this study, a field emission scanning electron microscope (FE-SEM, JSM-7100F) was used to analyze two-dimensional surface conditions and the chemical compositions (EDS) of biomedical grade NiTi wires (i.e., Ø 200 μm and Ø 400 μm) before and after polishing by an advanced magnetic abrasive finishing process. Before FE-SEM observations, the original sample and final polished sample were washed with an ultrasonic cleaner for 3 min in order to remove impurities from their surface. In addition, FE-SEM observation was carried out to evaluate the effects of surface roughness on bacterial adhesion (i.e., *E. coli* and *S. aureus*) on the surface of NiTi wire samples. The original sample and the final polished sample incubated with *E. coli* and *S. aureus* were prepared, and they were dehydrated using different concentrations of ethanol (i.e., 20, 30, 50, 80, and 100%) for 15 min each in order to be observed with FE-SEM. The number of bacteria adhered to the rough and smooth surface of NiTi wires was counted by the FE-SEM observation images, and they were calculated per square micrometer (µm²) of the surface area.

## 3. Results and Discussion

### 3.1. The Influence of Rotational Speed

**Figure 5:**
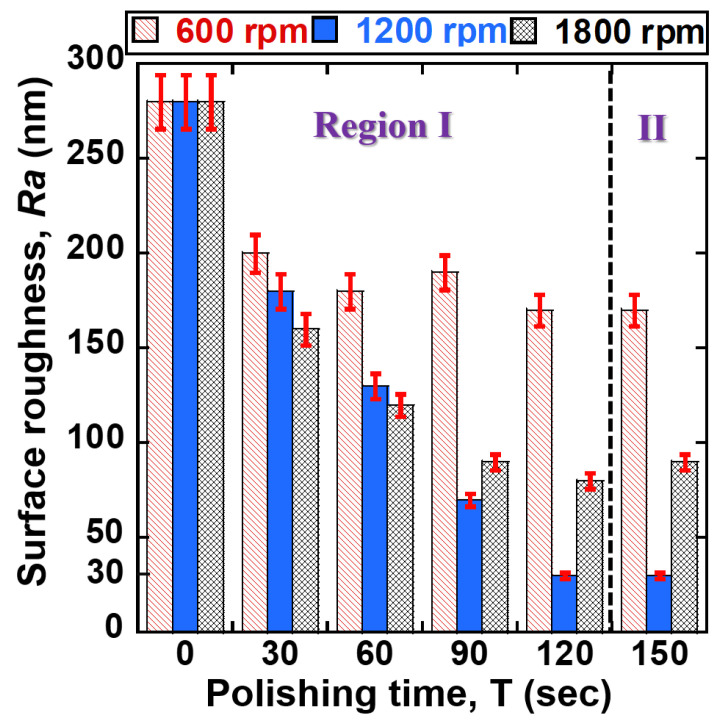
Correlation between surface roughness of 400 μm NiTi wire and polishing time at different rotational speeds (600, 1200, 1800 rpm).

**Figure 6:**
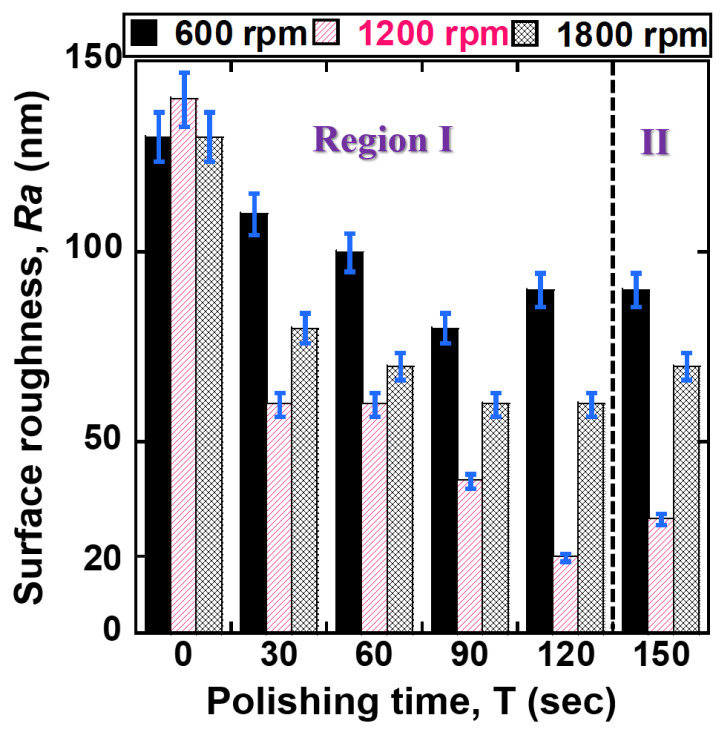
Correlation between surface roughness of 200 μm NiTi wire and polishing time at different rotational speeds (600, 1200, 1800 rpm).

Figure 5 illustrates the correlation between surface roughness *Ra* of 400 μm NiTi wire and polishing time at dissimilar rotational speeds (600, 1200, 1800 rpm). As illustrated in Figure 5, within 60 s of the polishing time, the values of *Ra* rapidly reduced with an increase in rotational speed. The *Ra* values of NiTi wire were reduced from original values of 280 nm to 180 nm, 130 nm, and 120 nm at the rotational speeds of 600, 1200, and 1800 rpm, respectively. When increased the polishing time to 120 s, the *Ra* values decreased to 170 nm, 30 nm, and 80 nm at 600 rpm, 1200 rpm, and 1800 rpm, respectively. However, within 150 s of polishing time, the *Ra* values of NiTi were not reduced further under all conditions (600 rpm, 1200 rpm, and 1800 rpm). Within 120 s of polishing, the 1200 rpm showed the lowest surface roughness compared to rotational speeds of 600 rpm and 1800 rpm. Figure 6 illustrates the correlation between the surface roughness of 200 μm NiTi wire and polishing time at different rotational speeds. Similarly, the surface roughness of 200 μm NiTi wire was reduced under all rotational conditions (600 rpm, 1200 rpm, and 1800 rpm). Among three conditions, 1200 rpm was the optimal rotational speed, followed by 1800 rpm and 600 rpm. Within 120 s of polishing time, the *Ra* values of 200 μm NiTi wires were reduced from their original 130–140 nm to 90 nm, 20 nm, and 60 nm at 600, 1200, and 1800 rpm, respectively. The polishing characteristics of each rotational speed on reducing the *Ra* values of 400 μm and 200 μm NiTi wire can be described as follows. As illustrated in Figure 5 and Figure 6, the characteristics of the reduction in surface roughness *Ra* are divided into two regions (I and II). The surface roughness is continuously reduced in the region I from 0 s to 120 s of polishing time, and then it undergoes a negligible reduction in region II after 120 s of polishing time. According to the results from Figure 5 and Figure 6, the higher rotational speeds (1200 rpm and 1800 rpm) can achieve better results in surface roughness reduction compared to the lower rotational speed of 600 rpm. The reason might be the greater unevenness of material removal from the surface of the NiTi wire when the magnetic micro-abrasive polishing (MMAP) rotates at a higher rotational speed, and the cutting actions of the abrasives on the surface of the NiTi wire were increased. For this reason, a smooth surface of NiTi stent wire could be achieved.

Surprisingly, when comparing two higher rotational speeds (i.e., 1200 rpm and 1800 rpm), 1200 rpm has better results in reducing surface roughness than 1800 rpm. This is due to the effects of other factors involved with higher rotational speed during the advanced MAF process, resulting in a reverse in the results between 1200 rpm and 1800 rpm. When the revolution speed is increased up to 1800 rpm, it is challenging to reduce the surface roughness to 0.08 μm. This might be explained by high centrifugal force and run-out of MMAP particles with increasing rotational speed up to 1800 rpm. The occurrence of run-out and high centrifugal force of MMAP causes strong collisions between the polishing abrasives and the surface of the NiTi workpiece, resulting in poor surface quality. The *Ra* values of the 400 and 200 μm NiTi wires did not continue to experience reductions in region II because the high peaks and unevenness were completely removed from the original surface of NiTi during the polishing process in region I. Thus, the rotational speed of 1200 rpm and polishing time of 120 s is optimal for achieving NiTi wire nano-scale surface roughness.

### 3.2. The Influence of PCD Grain Size

**Figure 7:**
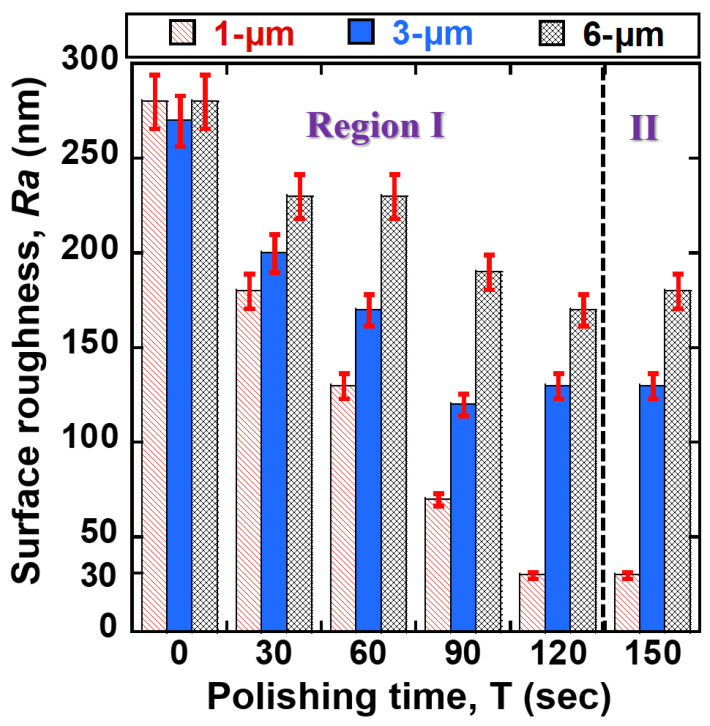
Correlation between surface roughness of 400 μm NiTi wire and polishing time according to abrasive grain sizes (1, 3, and 6 μm).

**Figure 8:**
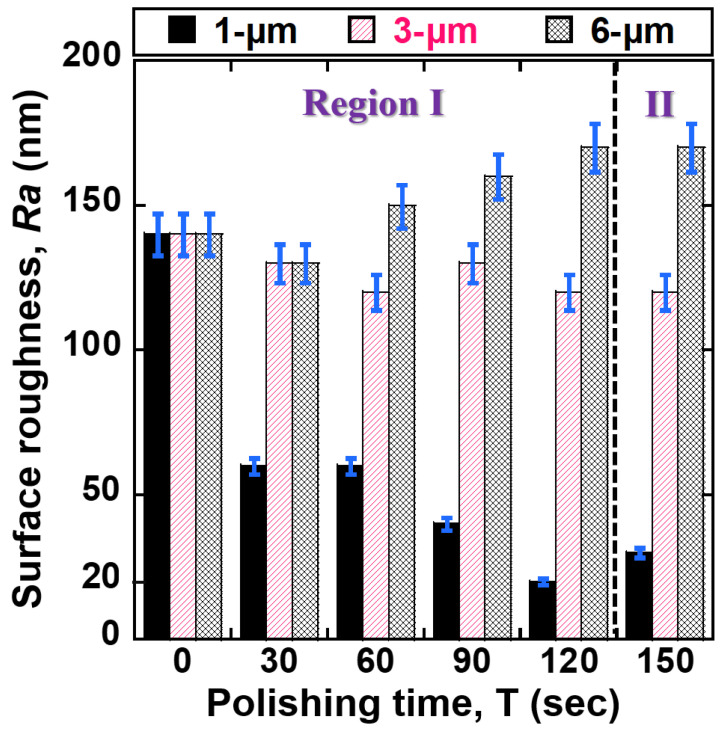
Correlation between surface roughness of 200 μm NiTi wire and polishing time conforming to abrasive grain sizes (1, 3, and 6 μm).

Figure 7 illustrates the correlation between surface roughness *Ra* of 400 μm NiTi wire and depending on processing time according to abrasive grain sizes of PCD micro-tools (1, 3, and 6 μm). The *Ra* values of 400 μm NiTi wire were reduced with all grain sizes of PCD micro-tools. The smallest reduction in *Ra* value was observed when a 1-μm grain size was used. Within 120 s of polishing time (see Region I), the *Ra* values of 400 μm NiTi were reduced from 280, 270, and 280 nm to 30, 130, and 170 nm by 1, 3, and 6 μm abrasive grain sizes, respectively. According to the results, the decreasing grain size of PCD abrasive to 1-μm can significantly reduce the *Ra* value of the sample, resulting in a finer surface polish. This can be explained by various polishing characteristics of each grain size of the PCD micro-tools. The bigger grain size of PCD produces a larger cutting edge compared to the smaller grain size. Larger cutting edges (6 and 3 μm) can remove more materials from the surface of NiTi wire than for the 1-μm case. However, the scratches made by these bigger cutting edges (6 and 3 μm) are bigger than those for the smaller cutting edge. Therefore, smaller abrasive particles produce better surface quality. Figure 8 illustrates the correlation between the surface roughness of 200 μm NiTi wire and polishing time. The original *Ra* values of the 200 μm NiTi wires were polished with different abrasive grain sizes (1, 3, 6 μm) for 150 s. Within 120 s of polishing (see Region I), the *Ra* values were reduced to 20 nm and 120 nm by 1 μm and 3 μm sizes, respectively. In contrast, the *Ra* value of the 200 μm NiTi wire was not reduced by 6 μm after 60 s of polishing time, and this trend continued until the end of polishing. Thus, when the original *Ra* value is less than 1400–1500 nm, it is difficult to reduce *Ra* with the larger 6-μm grain size. This is because the 1500 nm wide scratches on the surface of NiTi wire were created by the 6-μm particles. In region II, after 120 s until 150 s of the polishing time, the *Ra* values for both cases of NiTi wires (i.e., Ø 200 μm and Ø 400 μm) were not reduced further. This is because the inequality and high peaks were already removed from the surface of NiTi wires in region I. Thus, PCD micro-tools could not continue to significantly decrease the surface roughness of NiTi wires in region II. Therefore, polishing conditions of rotational speed: 1200 rpm, PCD micro-tools grain size: 1 μm, and polishing time: 120 s were optimal for reducing the surface roughness of NiTi wires to the nano-level.

### 3.3. FE-SEM Morphologies

**Figure 9:**
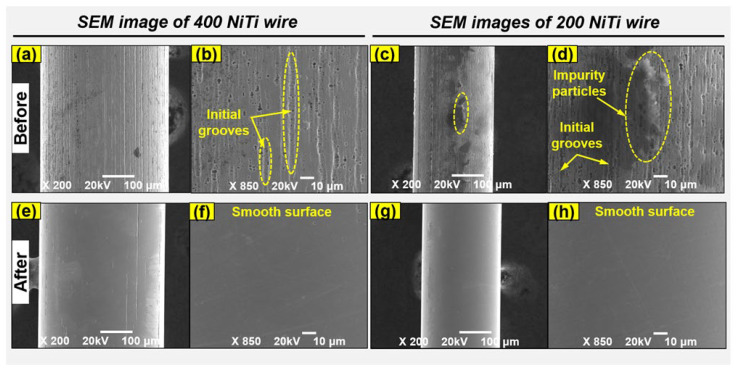
Surface conditions before and after processing: (**a**–**d**) the original and (**e**–**h**) final surface conditions of 400 and 200 μm NiTi wires at magnifications of ×200 and ×850.

Figure 9 illustrates the FE-SEM morphologies of surfaces of the 400 and 200 μm NiTi wires before and after polishing under the optimal conditions using the advanced MAF process. Figure 9a,b, respectively, show the original surface conditions of 400 μm NiTi wire at magnifications of ×200 and ×850. Figure 9c,d, respectively, show the original surface conditions of the 200 μm NiTi wire at magnifications of ×200 and ×850. Before polishing, multiple long grooves and micro holes with irregular shapes were present across the entirety of the original surfaces of the 400 and 200 μm NiTi samples. In addition, there were impurity particles present on the surfaces of wire materials (see Figure 9c,d).

These particles can affect the material properties of NiTi wire materials. After polishing at optimal conditions, the multiple long grooves and micro holes were mostly removed from the original surfaces of the 400 and 200 μm NiTi wire materials. The final surfaces of the 400 and 200 μm NiTi wires were smoother and cleaner compared to the original surfaces (see Figure 9a–d). Therefore, the MAF process using this nanoscale polishing technique enables smooth and clean NiTi wire surfaces, and the surface roughness was reduced to nanoscale dimensions of 30 nm for 400 μm NiTi and 20 nm for 200 μm NiTi wire material.

**Figure 10:**
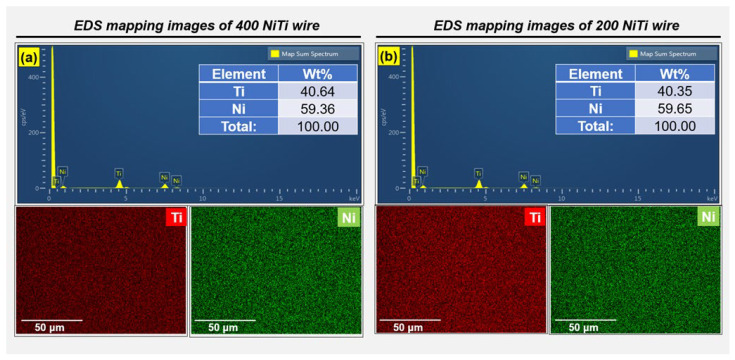
Elemental mapping analysis of a typical selected area on the surfaces of 400 and 200 μm NiTi wire materials: (**a**) in the case of 400 μm NiTi wire, (**b**) 200 μm NiTi wire.

The surfaces of NiTi wire materials need to be cleaned well and any toxic materials removed. Therefore, elemental mapping (EDS) is employed to analyze and characterize the chemical composition and toxicity on the surfaces of 400 and 200 μm NiTi wire materials before and after polishing. Figure 10 illustrates the elemental mapping analysis of a typical selected area on the surfaces of 400 and 200 μm NiTi wire materials. This verifies that NiTi wire samples contained Ni and Ti. After polishing the 400 μm NiTi wire, 40.64% Ti and 59.36% Ni were detected at the material surface. For the 200 μm NiTi wire, 40.35% Ti and 59.65% Ni were detected on the material surface. Compared to the elements of pre-polished 400 μm and 200 μm NiTi (see Table 1), there was no change in the elements of the NiTi wire caused by the advanced MAF process.

**Figure 11:**
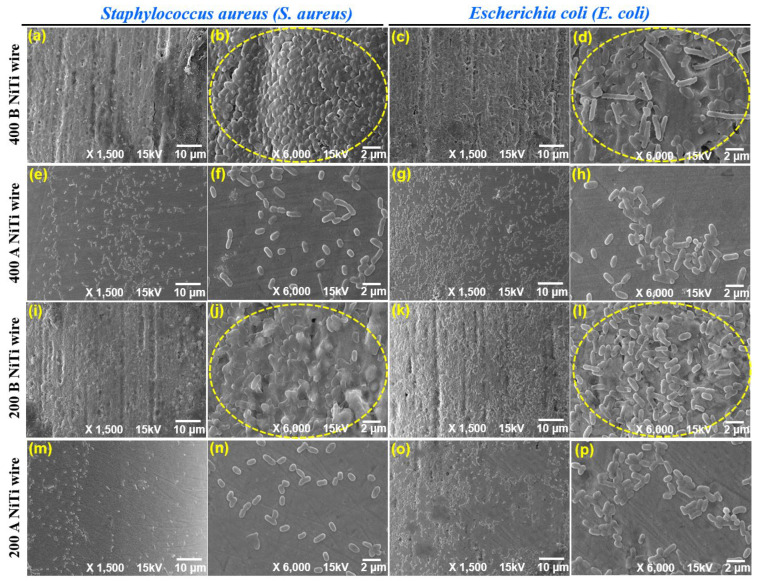
Representative FE-SEM images of (**a**,**b**) *S. aureus* and (**c**,**d**) *E. coli* adhered to the original surface of 400 μm NiTi, (**e**,**f**) *S. aureus* and (**g**,**h**) *E. coli* adhered to the polished surface of 400 μm NiTi, (**i**,**j**) *S. aureus* and (**k**,**l**) *E. coli* adhered to the original surface of 200 μm NiTi, (**m**,**n**) *S. aureus* and (**o**,**p**) *E. coli* adhered to the polished surface of 200 μm NiTi at magnifications of ×1500 and ×6000.

In this study, FE-SEM images are used for investigating and evaluating bacterial adhesion to different surfaces of NiTi wires. Figure 11 shows FE-SEM images of the bacterial adhesion (i.e., *Escherichia coli* (*E. coli*) and *Staphylococcus aureus* (*S. aureus*)) on the surfaces of the 400 μm and 200 μm NiTi wire materials. Figure 11a–d, respectively, show *S. aureus* and *E. coli* adhesion on the original surface of the 400 μm NiTi wires before the advanced MAF process. Figure 11i–l are FE-SEM images of 200 μm NiTi wires before the advanced MAF process. As shown, the surfaces of NiTi wire differed before treatment by an advanced MAF, and bacterial adhesion was promoted by the micro/nano-scale patterns. Before MAF, the NiTi wire has micron-scale longitudinal-direction defect sites on the surface. The surfaces of the 400 μm and 200 μm NiTi wires were completely covered by bacteria strongly bound to the grooves on the original surfaces. Vast quantities of bacteria were attached to the surfaces of the samples. Figure 11e–h,m–p, are the results of bacterial adhesion to the final polished surfaces of the 400 μm and 200 μm NiTi wires, respectively. In contrast to the original surfaces, bacteria cultured on the polished NiTi wires (400 μm and 200 μm) showed a small number of colonies. Generally, biofilm formation progresses following the formation of bacterial clusters and microcolonies. In this study, the as-received 400 μm and 200 μm NiTi wires showed the fast formation of bacterial clusters. It was concluded that the smoothness and cleanliness of wire surfaces with nanoscale smoothness through an advanced MAF process reduce the possibility of bacteria adhering, which is expected to decrease the rate of biofilm formation.

The percentage reduction in bacteria (i.e., *E. coli* and *S. aureus*) adhered to the surface of the NiTi wire material can be expressed by Equation (1). NBAR (Number of bacteria adhered to roughness) is the number of bacteria adhered to the rough surface of NiTi wire before nanoscale polishing, and NBAS (Number of bacteria adhered to smoothness) is the number of bacteria adhered to the smooth surface of the NiTi wire after polishing, which is calculated within 120 s of polishing time. PRBA (%) is a percentage reduction in the bacteria adhered to the surface of the NiTi wire. According to Equation (1), within 120 s of polishing time, the percentage reduction in bacterial adhesion for *Staphylococcus aureus* was about 83.48%, while *Escherichia coli* was about 70.67%. This indicates that the percentage reduction in the bacteria adhered to the surface of the NiTi wire was more than 70%.

This result demonstrates the ability of an advanced MAF nanoscale polishing technique as an affordable and feasible means to reduce the surface roughness of biomedical grade NiTi wires to reduce bacterial adhesion, likely resulting in a dramatic reduction in implant-related infections.

(1) $$PRBA\left(\%\right)=\frac{NBAR\left(bac/\mu m^{2}\right)-NBAS\left(bac/\mu m^{2}\right)}{NBAR\left(bac/\mu m^{2}\right)}\times 100$$

## 4. Conclusions

In this study, an advanced magnetic abrasive finishing (MAF) process using a nanoscale polishing technique was used to polish the surfaces of biomedical grade NiTi wire materials with different diameters (i.e., Ø 200 μm and Ø 400 μm). The bacterial adhesion tests confirmed the effects of bacterial adhesion on NiTi wire through treatment with the advanced MAF process. The conclusions of this study can be summarized as follows:

1. The experimental results revealed that the original surface of 200 μm and 400 μm NiTi wires were achieved to an ultra-smooth surface polish by an advanced MAF process. In the case of 200 μm NiTi wire, the *Ra* values of 200 μm NiTi wires were smoothly improved from 130–140 nm to 90, 20, and 60 nm at 600, 1200, and 1800 rpm of rotational speed with 120 s of polishing time. While in the case of 400 μm NiTi wire, the *Ra* values of 400 μm NiTi wires were smoothly improved from 280 nm to 170, 30, and 80 nm at 600, 1200, and 1800 rpm. This indicates that the proposed process is desirable for polishing biomedical materials to the nanoscale level.
2. Under the optimal conditions of the MAF process, the percentage reduction in bacterial adhesion for *Staphylococcus aureus* was about 83.48%, while *Escherichia coli* was about 70.67%. This indicates that the percentage reduction in the bacteria adhered to the surface of the NiTi wire was more than 70%.
3. According to the results, the different input process parameters significantly affect the ability of the advanced MAF process to achieve smooth surfaces of NiTi wire materials. When a smaller grain size (1-μm) was used, a very smooth surface was achieved. However, when larger grain sizes (3, 6-μm) were used, it was difficult to reduce the surface roughness *Ra* values of wire materials, resulting in values in the range of 140–150 nm. This is because the larger grain sizes of abrasive themselves scratch the surface of NiTi wire materials when the polishing time is lengthened.
4. A rotational speed of 1200 rpm achieved better surface roughness reduction compared to a lower rotational speed of 600 rpm. However, by further increasing the rotational speed to 1800 rpm, the surface roughness quality of the NiTi wire materials was not significantly reduced. Further increases in rotation speed could lead to increased centrifugal force and run-out of the abrasive tools, resulting in increased difficulty in achieving smooth surfaces of the NiTi wires.
5. FE-SEM images clearly showed the smoothness and cleanliness of the surfaces of NiTi wire materials achieved by this advanced MAF process using the nanoscale polishing technique.
6. The bacterial adhesion study revealed that bacterial adhesion has a strong relationship with the surface roughness of biomedical-grade NiTi wires. By reducing the surface roughness to the nano-scale, the ability of bacteria to adhere is decreased, likely resulting in a dramatic reduction in implant-related infections.
7. In future work, the effect of surface roughness on shape memory alloy self-expanding NiTi stent on the initial cell adhesion will be studied. Crystal violet staining technique will be carried out for the NiTi stent sample with smooth and rough sample surfaces after 1.5 h cell incubation.

## Figures and Tables

**Table 1:** Chemical compositions of Ø 400 μm and Ø 200 μm NiTi wires in weight percentage (Wt.%).

No.	Element	Ni	Ti	Total
NiTi wire (Ø 400 μm)	Chemical composition	58.28	41.72	100
NiTi wire (Ø 200 μm)	Chemical composition	58.66	41.34	100

**Table 2:** Experimental conditions.

Parameter	Value
Samples	Biomedical grade NiTi wires (dimension: Ø 200 μm, Ø 400 μm)
Polishing abrasive tools	Fe micron powder (Fe#200): 0.8 g PCD micro-tools: 1.0 g Grape seed oil (GSO): 400 µL
PCD micro-tools	Grain size: 1, 3, 6 μm
Rotational speeds	600, 1200, 1800 rpm
Finishing gap	5 mm
Vibration of polishing tools	Frequency (f): 10-Hz Amplitude (a): 3 mm
Type of magnet	Neodymium magnet (Nd-Fe-B)
Polishing times	0, 30, 60, 90, 120, 150 s

## Data Availability

The data presented in this study are available on request from the corresponding author.
